# Expression of Concern for Galdiero et al., “*Haemophilus influenzae* Porin Contributes to Signaling of the Inflammatory Cascade in Rat Brain”

**DOI:** 10.1128/iai.00490-24

**Published:** 2024-11-29

**Authors:** American Society for Microbiology

## EXPRESSION OF CONCERN

The American Society for Microbiology (ASM) and *Infection and Immunity* (IAI) are issuing this Expression of Concern regarding the following publication:

Galdiero M, D'Amico M, Gorga F, Di Filippo C, D'Isanto M, Vitiello M, Longanella A, Tortora A. 2001. *Haemophilus influenzae* porin contributes to signaling of the inflammatory cascade in rat brain. Infect Immun 69:221–227. https://doi.org/10.1128/iai.69.1.221-227.2001.

ASM and IAI were notified of concerns that the agarose gel for β-actin presented in Fig. 2A and the agarose gel for β-actin presented in Fig. 3A are likely identical.

ASM further verified these concerns. This Expression of Concern is issued to raise awareness of the above-mentioned concerns and will be updated accordingly if new information becomes available. In publishing this Expression of Concern, ASM is following its Publishing Ethics Policies and Procedures and, as a member of the Committee on Publication Ethics (COPE), follows its guidelines in this regard.

